# Blue carbon stores in tropical seagrass meadows maintained under green turtle grazing

**DOI:** 10.1038/s41598-017-13142-4

**Published:** 2017-10-19

**Authors:** Robert A. Johnson, Alexandra G. Gulick, Alan B. Bolten, Karen A. Bjorndal

**Affiliations:** Archie Carr Center for Sea Turtle Research and Department of Biology, University of Florida, Gainesville, FL 32611 USA

## Abstract

Seagrass meadows are important sites for carbon storage. Green turtles (*Chelonia mydas*) are marine megaherbivores that consume seagrass throughout much of their global range. With successful conservation efforts, turtle abundance will increase, leading to more meadows being returned to their natural grazed state. There is concern this may lead to a loss of carbon stored in these systems, but the effects of green turtle grazing on seagrass ecosystem carbon dynamics have not been investigated. Here we experimentally show that despite 79% lower net ecosystem production (NEP) following grazing (24.7 vs. 119.5 mmol C m^−2^ d^−1^) in a Caribbean *Thalassia testudinum* seagrass meadow, grazed areas maintained net positive metabolic carbon uptake. Additionally, grazing did not change the meadow production to respiration ratio, indicating it did not stimulate remineralization of sediment carbon stores. Compared to other published estimates of seagrass NEP (median: 20.6 mmol C m^−2^ d^−1^), NEP in grazed Caribbean *T. testudinum* meadows is similar to that in many other ungrazed systems. Our results demonstrate that while grazing does decrease potential future carbon sequestration as a result of lower NEP, it does not promote a metabolic release of current carbon stocks.

## Introduction

Seagrass meadows form some of the most productive ecosystems in the world^1^. Seagrasses sequester large amounts of ‘blue carbon’—carbon buried by vegetated marine systems—each year through high rates of production and organic matter burial^2–4^. The majority of this carbon is stored belowground in the sediments, where anoxic conditions can result in storage for millennia^5^. This suggests that conservation and restoration of seagrass systems could be used as a climate change mitigation strategy^6–8^.

**Figure 1:**
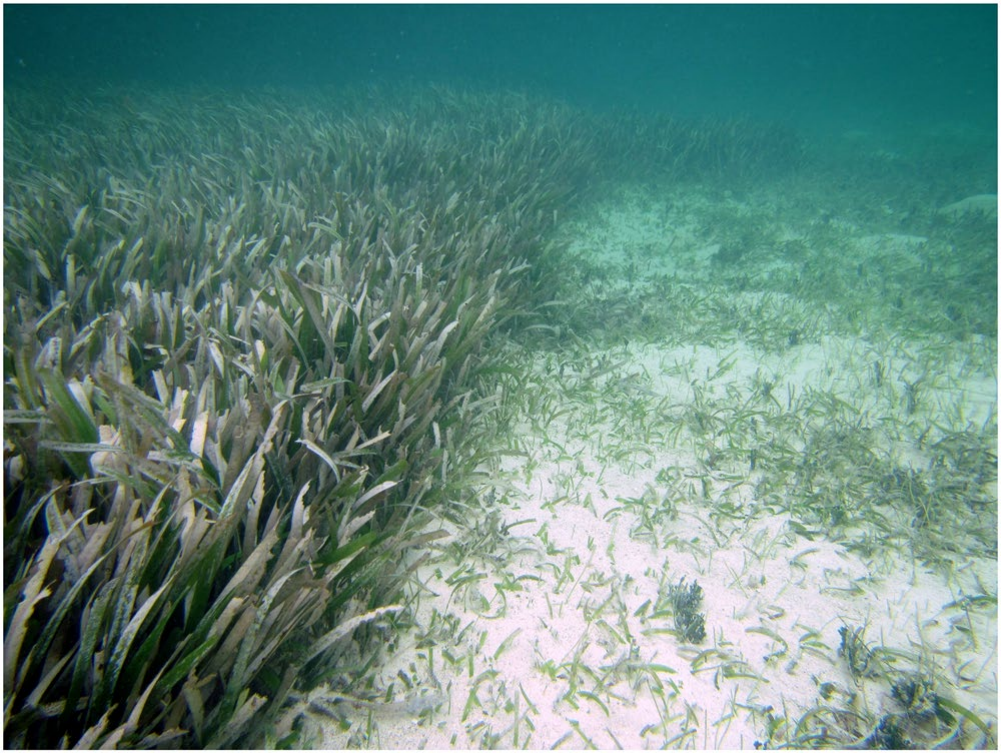
A naturally grazed green turtle feeding plot (right) and an adjacent ungrazed area (left) in a *Thalassia testudinum* seagrass meadow in Little Cayman. Photo by Robert A. Johnson.

Green turtles (*Chelonia mydas*) are megaherbivores that consume seagrass as a large part of their diet across much of their global range. Green turtles establish feeding plots in which they forage by cropping seagrass blades at or near the sediment surface (Fig. 1) and repeatedly re-grazing new growth within these plots^9^, thereby structurally altering the meadow^10^. With successful conservation leading to increasing green turtle populations in some areas^11^, seagrasses will increasingly be subjected to grazing pressure in addition to anthropogenic disturbances^12–15^.

Supporting abundant green turtle populations and sustaining a carbon sink are both important conservation aims for global seagrass ecosystems. Grazed meadows with intact grazer populations is the natural state of seagrass ecosystems. Historically, when green turtle abundance was much higher than today^16,17^, Caribbean seagrass meadows supported extensive grazing^18^, with the majority of meadows likely in a grazed state before humans disrupted these coevolved systems through overexploitation of green turtles^16,19^. While green turtle grazing helps maintain meadow health by removing older, pathogen-susceptible seagrass blades^9,16^, grazing also reduces the size of the photosynthetic seagrass canopy that is capable of allocating production belowground for storage^9,20,21^. It has been hypothesized that grazing may have negative effects on seagrass meadow carbon sequestration and storage, and that the conservation of both green turtles and seagrass carbon stores are incompatible^22^.

To better conserve these ecosystems, it is necessary to understand how ecosystem processes, such as carbon sequestration, operate within seagrass meadows in their naturally grazed state. We hypothesized that: (1) metabolic carbon uptake rates (net ecosystem production) would be lower in grazed compared to ungrazed areas as a result of reduced aboveground biomass, and (2) carbon remineralization rates (ecosystem respiration) would be proportionally higher in grazed than in ungrazed areas as a result of increased heterotrophic respiration due to aeration of surface sediments following removal of the seagrass canopy.

We conducted an experimental manipulation in Little Cayman, Cayman Islands, by clipping seagrass (*Thalassia testudinum*) to simulate green turtle grazing. We measured areal rates of gross primary production (GPP), ecosystem respiration (R_E_), and net ecosystem production (NEP = GPP–R_E_) weekly with benthic incubation chambers in five experimentally clipped plots and five unclipped reference plots to investigate changes in carbon dynamics following the onset of simulated grazing. Metabolism (GPP, R_E_, NEP) was similarly measured in nearby areas that were naturally, actively grazed for at least a year by juvenile green turtles and adjacent ungrazed areas, and these results were compared to those from the experimental and reference plots. To evaluate our measured rates and the effects of green turtle grazing in a broader geographical context, we also compiled published estimates of seagrass ecosystem metabolism.

## Results

### Effect of Grazing on Seagrass Ecosystem Metabolism

**Figure 2:**
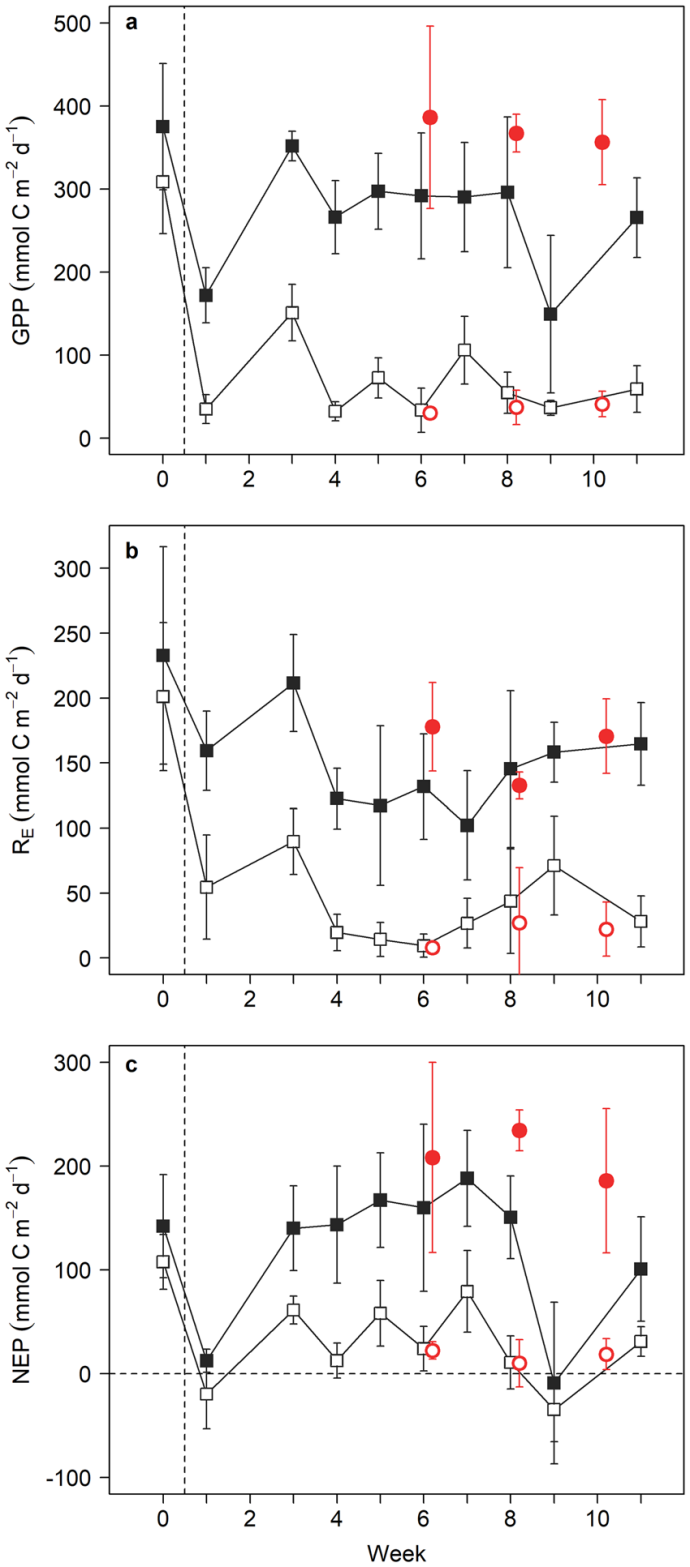
Daily metabolic rates following simulated or natural grazing. (**a**) Gross primary production. (**b**) Ecosystem respiration. (**c**) Net ecosystem production. Data are means (±SD). Open black squares are clipped plots, closed black squares are reference plots, open red circles are naturally grazed areas, and closed red circles are ungrazed areas. Week 0 began on 15 May 2016, and week 11 began on 31 July 2016. Vertical dashed line denotes initiation of clipping. Horizontal dashed line in (**c)** denotes metabolic balance (NEP = 0). See text for description of fluctuating clipped plot values.

Ecosystem metabolism (GPP, R_E_, NEP) was significantly lower in experimentally clipped plots compared to reference plots (mixed effects model; GPP, n = 83, F_1,8_ = 106.4, p < 0.0001; R_E_, n = 83, F_1,8_ = 75.3, p < 0.0001; NEP, n = 83, F_1,8_ = 34.8, p = 0.0004; Table 1). This difference persisted for the duration of the experimental manipulation (Fig. 2). Prior to the onset of clipping there were no differences in measured metabolic rates between clipped and unclipped reference plots (t-test; GPP, n = 8, t_6_ = −1.3, p = 0.23; R_E_, n = 8, t_6_ = −0.6, p = 0.55; NEP, n = 8, t_6_ = −1.2, p = 0.27), and all plots were autotrophic (NEP > 0). During the experiment, GPP was 77% lower, R_E_ 74% lower, and NEP 79% lower in clipped plots on average compared to reference plots (Table 1). Clipping reduced aboveground seagrass biomass by an average of 80%, but this was not enough to shift clipped plots from positive to negative NEP, and they remained metabolic carbon sinks (NEP > 0) for the duration of the 12-week experiment. The two occasions (weeks one and nine; Fig. 2) when clipped plots were slightly heterotrophic (NEP < 0) and reference plots were reduced to near metabolic balance (NEP = 0) was a result of low GPP when incubations were conducted on overcast days.

**Table 1:** Means and standard deviations of metabolic rates and seagrass parameters for clipped plots, reference plots, naturally grazed areas, and ungrazed areas.

**Variable**	**Clipped**	**Reference**	**Grazed**	**Ungrazed**	**Test**	**P-value**
GPP (mmol C m^−2^ d^−1^)	64.4 ± 40.4^**a**^	275.5 ± 69.9^**b**^	36.1 ± 5.4	370.0 ± 15.0	MEM	<0.0001
R_E_ (mmol C m^−2^ d^−1^)	39.7 ± 27.4^**a**^	154.6 ± 41.2^**b**^	19.1 ± 9.9	160.4 ± 24.3	MEM	<0.0001
NEP (mmol C m^−2^ d^−1^)	24.7 ± 37.6^**a**^	119.5 ± 66.2^**b**^	17.1 ± 6.4	209.5 ± 24.3	MEM	0.0004
P:R	2.3 ± 1.6^**a**^	1.9 ± 0.6^**a**^	2.4 ± 1.3^**a**^	2.3 ± 0.4^**a**^	ANOVA	0.16
Shoot Density (shoots m^−2^)	917.8 ± 85.1^**a**^	840.0 ± 40.0	776.4 ± 31.8^**a**^	785.8 ± 40.1	t-test	0.1408
Blade Length (cm)	4.8 ± 0.9^**a**^	15.8 ± 1.5	1.9 ± 0.4^**b**^	14.9 ± 2.8	t-test	0.0012
Blade Width (cm)	0.9 ± 0.1^**a**^	0.9 ± 0.04	0.5 ± 0.1^**b**^	1.0 ± 0.1	t-test	0.0004
AG Biomass (g DM m^−2^)	52.1 ± 16.5^**a**^	259.4 ± 44.6	8.6 ± 4.3^**b**^	193.8 ± 21.5	t-test	<0.0001
BG Biomass (g DM m^−2^)	3880.7 ± 1256.3^**a**^	3899.7 ± 1166.4^**a**^	3562.4 ± 1002.6^**a**^	4143.8 ± 961.7^**a**^	ANOVA	0.611

Within a row, values that share a letter superscript are not significantly different. GPP: gross primary production; R_E_: ecosystem respiration; NEP: net ecosystem production; P:R: production to respiration ratio; AG: aboveground; BG: belowground; MEM: mixed effects model.

We measured ecosystem metabolism using the same methods in naturally grazed areas and adjacent ungrazed areas of seagrass located near our experimental plots. These naturally grazed areas were actively maintained by juvenile green turtles and had been grazed continuously for at least one year. Net ecosystem production in naturally grazed areas was 92% lower than adjacent ungrazed areas on average, and we compared results from these naturally grazed areas, representing long-term effects of grazing, to those from our experimentally clipped plots, representing short-term effects of grazing. Metabolic rates in the naturally grazed areas were similar to those in our clipped plots (Fig. 2). This was unexpected given that aboveground biomass was significantly lower in the naturally grazed areas than our clipped plots (t-test; n = 9, t_7_ = 8.5, p < 0.0001; Table 1). While seagrass shoot density was not significantly different between naturally grazed areas and clipped plots, seagrass blades in the naturally grazed areas were significantly shorter (t-test; n = 9, t_7_ = 5.2, p = 0.0012) and narrower (t-test; n = 9, t_7_ = 6.3, p = 0.0004) than in the clipped plots yielding less photosynthetic leaf area (Table 1). Unlike certain seagrass parameters, such as shoot density and blade width, which may take months to become reduced following the onset of grazing^10^, seagrass ecosystem metabolism (GPP, R_E_, NEP) experienced a rapid reduction following the onset of simulated grazing, after which rates remained relatively stable (Fig. 2).

**Figure 3:**
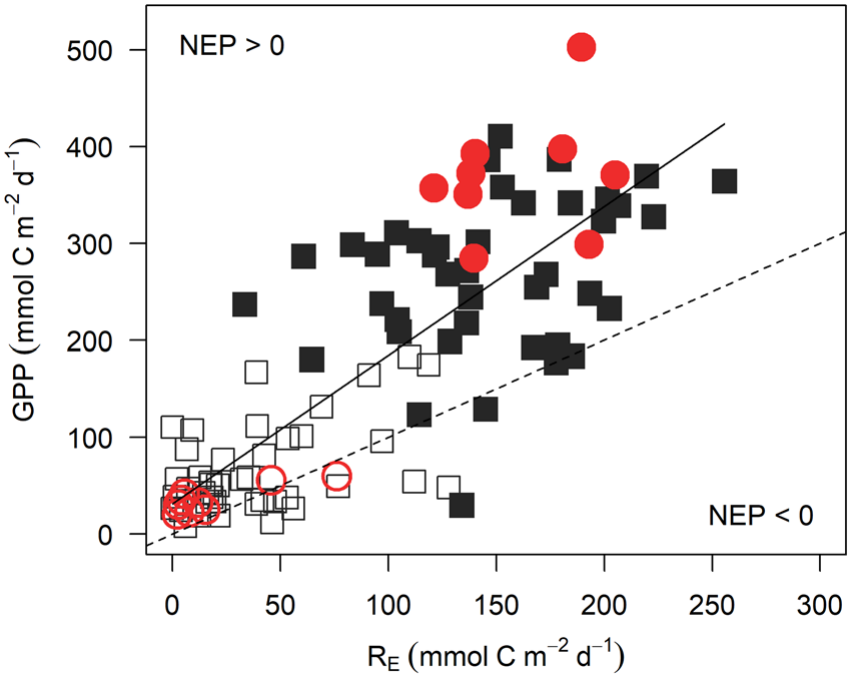
Production to respiration ratios. Data are from all plots on all sampling days. Open black squares are clipped plots, closed black squares are reference plots, open red circles are naturally grazed areas, and closed red circles are ungrazed areas. Solid line is the significant linear regression (R^2^ = 0.66, p < 0.0001). Dashed line is the 1:1 ratio (NEP = 0).

The relative contribution of GPP and R_E_ to the total metabolism of the ecosystem (measured by the production to respiration ratio, P:R) did not differ among treatments (clipped, reference, grazed, ungrazed; ANOVA; n = 99, F_3_ = 1.8, p = 0.16; Table 1), even though rates of GPP and R_E_ were lower in clipped plots and naturally grazed areas than unclipped reference plots and ungrazed areas. GPP was also strongly, positively correlated with R_E_ (linear regression; n = 101, R^2^ = 0.66, p < 0.0001; Fig. 3). High P:R ratios (range 1.9–2.4; Table 1) show the meadow was strongly autotrophic (NEP > 0) irrespective of green turtle grazing—natural or simulated.

### Role of Biomass in Carbon Uptake

**Figure 4:**
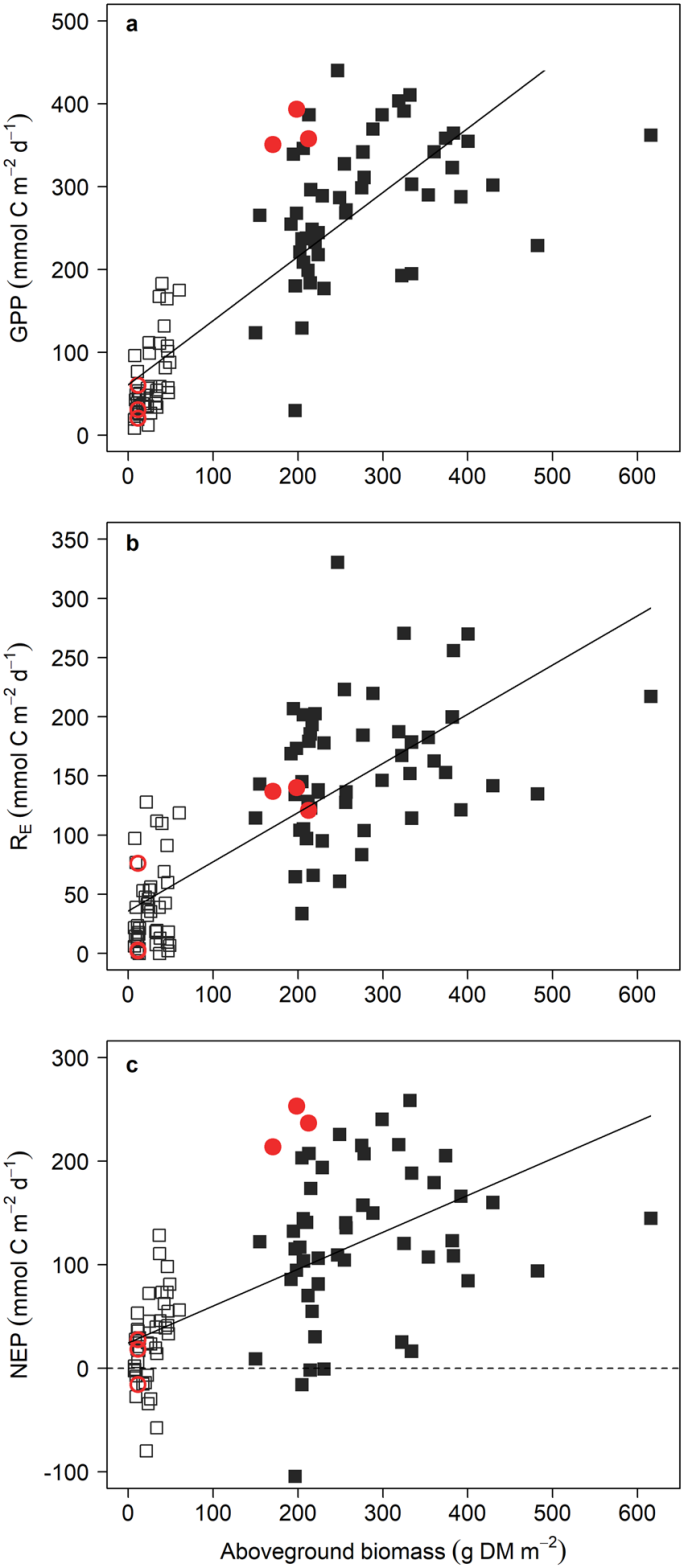
Metabolic rates as a function of aboveground seagrass biomass. (**a**) Gross primary production. (**b**) Ecosystem respiration. (**c**) Net ecosystem production. Open black squares are clipped plots, closed black squares are reference plots, open red circles are naturally grazed areas, and closed red circles are ungrazed areas. Solid lines are the significant linear regressions (GPP, R^2^ = 0.69, p < 0.0001; R_E_, R^2^ = 0.58, p < 0.0001; NEP, R^2^ = 0.37, p < 0.0001). Horizontal dashed line in (**c**) denotes metabolic balance (NEP = 0).

Aboveground seagrass biomass (dry mass; DM) was strongly and positively correlated with measures of ecosystem metabolism (linear regression; GPP, n = 96, p < 0.0001; R_E_, n = 96, p < 0.0001; NEP, n = 96, p < 0.0001; Fig. 4) and explained 69%, 58%, and 37% of the variability in GPP, R_E_, and NEP, respectively. Aboveground biomass fluctuated in clipped plots (range 0.0–72.9 g DM m^−2^) with the clipping regime while biomass in the reference plots remained relatively high (range 205.9–307.7 g DM m^−2^) during the experiment. Belowground biomass did not differ between clipped plots, reference plots, naturally grazed areas, or ungrazed areas (ANOVA; n = 34, F_2_ = 0.5, p = 0.611; Table 1).

### Grazing in a Global Context

**Figure 5:**
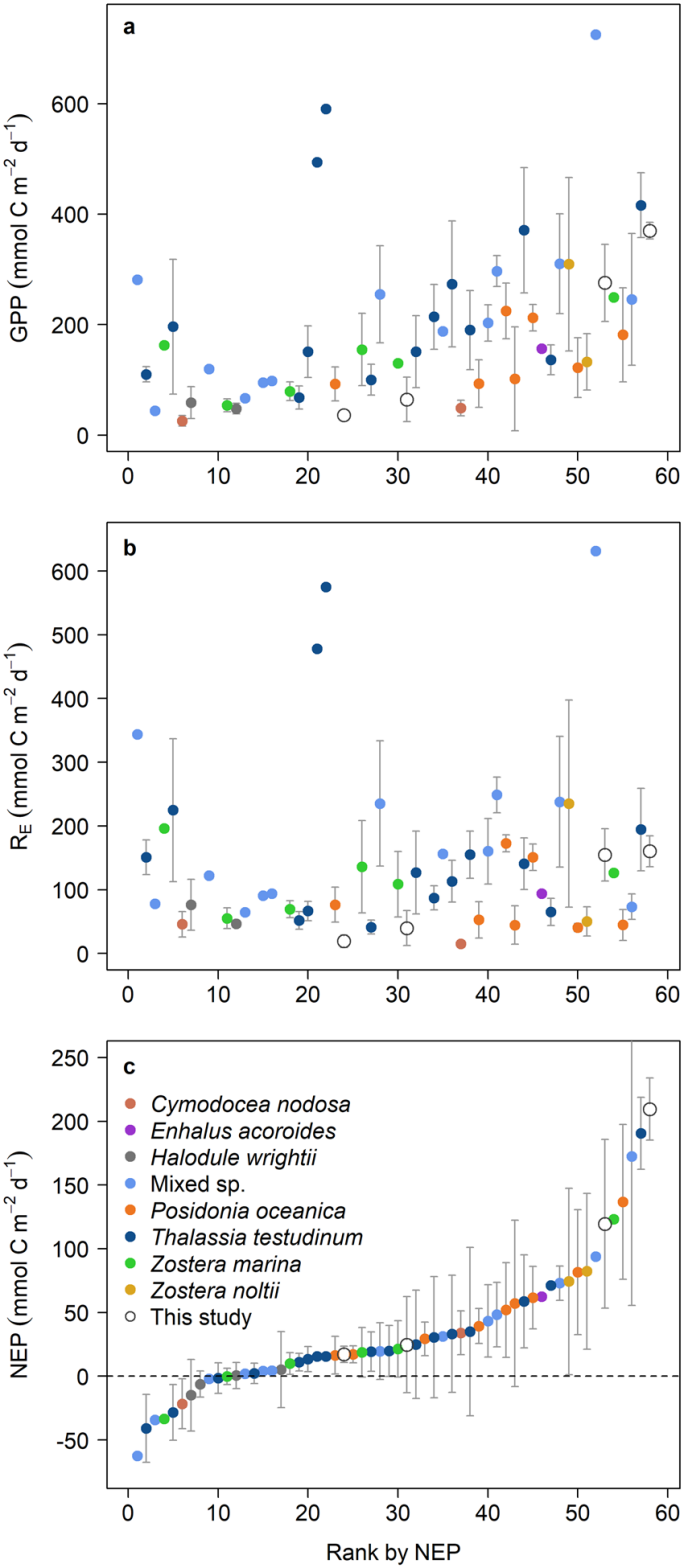
Seagrass ecosystem metabolism values compiled from the literature. (**a**) Gross primary production. (**b**) Ecosystem respiration. (**c**) Net ecosystem production. Data are means (±SD). Data from all studies (n = 58) are ranked by NEP. Open circles (this study) from left to right are: naturally grazed areas, clipped plots, reference plots, ungrazed areas (all panels). Horizontal dashed line in (**c**) denotes metabolic balance (NEP = 0).

To evaluate the results of our experiment from Little Cayman in a broader geographical context, we compiled estimates of seagrass metabolic rates (n = 58) from the published literature (see Supplementary Data 1). Reported rates of NEP from various seagrass species and areas around the world (including this study) ranged from −62.5 to 209.5 mmol C m^−2^ d^−1^ with a median of 20.6 (Fig. 5c). A majority of systems (81%) had net positive NEP, including those measured either from single sampling events or over the annual cycle. NEP in ungrazed *T. testudinum* meadows in Little Cayman (209.5 mmol C m^−2^ d^−1^) was higher than rates measured elsewhere, and NEP in our unclipped reference plots was also high, due to the high GPP relative to R_E_ in this system. Higher rates of both GPP and R_E_ have been measured in other seagrass systems than the rates we measured in Little Cayman (Fig. 5a,b), but the P:R ratio was always closer to one, resulting in lower rates of NEP for those systems^23–31^.

## Discussion

Despite significantly lower rates of carbon uptake (inferred from NEP), our results show that tropical *T. testudinum* meadows remained active metabolic carbon sinks, even under long-term sustained grazing pressure. Though green turtle grazing removes much of the aboveground seagrass biomass, the belowground biomass is normally left intact in *T. testudinum* meadows (see Table 1). Production may still be allocated to belowground tissues for long-term storage, thus allowing the meadow to remain a metabolic carbon sink in the presence of green turtle grazing. An example in Indonesia is an exception to this, in which green turtles became hyper-abundant (20 individuals ha^−1^) within a marine protected area and began digging and consuming belowground seagrass tissues (*Halodule uninervis*) when aboveground biomass alone was not enough to sustain the population^12^. The turtles may have been able to uproot and consume *H. uninervis* due to its shallow rhizosphere^32^ as compared to other seagrasses such as *T. testudinum* that form stronger and denser rhizome mats. To our knowledge, this behavior has not been observed elsewhere. In addition, green turtles often do not graze an entire meadow, but rather discrete patches^9,33,34^. Though NEP was 92% lower in areas that had been grazed long-term by turtles, the reduction in whole-meadow NEP would be less than this, as NEP in ungrazed areas remained unaffected. Changes in the strength of the ecosystem metabolic carbon sink from grazing are therefore dependent upon the proportion of grazed to ungrazed areas within the meadow.

The strong relationship between metabolic variables (GPP, R_E_, NEP) and aboveground seagrass biomass indicates that metabolic carbon dynamics in this system were largely driven by the seagrass rather than other potential producers such as epiphytes or microphytobenthos. This relationship also demonstrates that under a sustained green turtle grazing regime, rates of metabolic carbon uptake will remain lower than ungrazed areas. If turtles abandon or are excluded from an area, rates of carbon uptake will increase concomitantly with seagrass regrowth. This is further evidenced by fluctuations in NEP in our experimentally clipped plots. NEP was lower (15.8 ± 7.2 mmol C m^−2^ d^−1^) when measured 2–3 days after plots were clipped and aboveground biomass was low (10.5 ± 0.8 g DM m^−2^; even-numbered weeks; Fig. 2c), and higher (57.4 ± 19.9 mmol C m^−2^ d^−1^) when measured 8–10 days post-clipping and biomass had increased (33.9 ± 9.9 g DM m^−2^; odd-numbered weeks).

Our results show that meadows grazed by green turtles can maintain net positive metabolic carbon uptake, but there is concern as to what effect recovering green turtle populations—and increased grazing—will have on current seagrass blue carbon stocks^22^. It has been suggested that following extreme seagrass degradation or loss, such as from overgrazing, carbon stored in the top meter of sediment may be vulnerable to remineralization and loss from the system^4,35^. The production to respiration ratio (P:R) of the system can be used to investigate this indirectly, where increased microbial activity and organic carbon remineralization can be inferred from a decrease in the ratio.

Ecosystem respiration includes both respiration by the autotroph community (R_A_) and respiration by the heterotroph community (R_H_). Sediments in seagrass meadows are often anoxic below the first few millimeters to centimeters^7,36^, and we had predicted microbial activity and R_H_ would increase following removal of the seagrass canopy due to increased water flow and aeration of the surface sediments. An increase in R_H_ relative to R_A_ would increase R_E_ relative to GPP and therefore decrease the P:R ratio. That the P:R ratio was not affected by short-term experimental clipping or long-term natural grazing indicates that rates of R_E_ were largely driven by the amount of aboveground seagrass biomass present rather than by R_H_ in the benthos.

Anaerobic metabolism and subsurface carbon dynamics can also play a role in seagrass meadow carbon cycling. We were unable to measure these processes in our study; however, we feel that these were likely to play a small role in total carbon dynamics in this system. Following a decrease in primary production and oxygen translocation to the rhizosphere, organic matter remineralization typically switches from aerobic respiration to sulfate reduction^37^; however, rates of sulfate reduction are known to be low in carbonate-based sediments^38^, such as those in Little Cayman. Additionally, carbon dioxide produced from belowground remineralization of organic carbon may be consumed via carbonate dissolution, rather than released from the system^38,39^.

Grazing does lead to a loss of potential future blue carbon sequestration (carbon that may have become sequestered) as a result of lower NEP in grazed areas compared to ungrazed areas, but it did not affect the ecosystem P:R ratio. The meadow maintained net positive carbon uptake and high GPP relative to R_E_, indicating that grazing is not likely to lead to the metabolic release of blue carbon already stored in tropical *T. testudinum* meadows, and these carbon stocks may remain intact in the face of increasing grazing pressure. A similar relationship between grazing and sediment carbon has been shown in a Canadian salt marsh—areas that had been grazed long-term by sheep exhibited higher soil organic carbon content as well as higher belowground biomass production than ungrazed areas^40^. While seagrass meadows are resilient to long-term grazing^10^, the resilience of meadows to external stressors (e.g. decreased light availability from coastal runoff and sedimentation/eutrophication) may differ between grazed and ungrazed areas experiencing high stress levels. Future research into the effects of grazing in meadows experiencing high stress levels, such as from reduced light availability, would be beneficial in furthering our understanding of the ecological effects of green turtle grazing.

In comparison with seagrass metabolic values from the literature (Fig. 5), rates of GPP in our experimentally clipped plots and the naturally grazed areas are low (among studies that met our criteria for inclusion, see Methods; Fig. 5a). However, the high rates of GPP relative to R_E_ in our system resulted in higher NEP than that reported for many other seagrass systems (Fig. 5c). Some of these previously published rates could be low due to methodological reasons, as incubation time has been shown to influence measured metabolic rates^41^ (see Methods). NEP in both our clipped plots and naturally grazed areas (24.7 and 17.1 mmol C m^−2^ d^−1^, respectively) was near the median (20.6 mmol C m^−2^ d^−1^) of seagrass ecosystem metabolism estimates compiled from the literature. This suggests that even under a scenario of increasing green turtle grazing pressure, Caribbean *T. testudinum* meadows could still function as a stronger metabolic carbon sink than some ungrazed seagrass meadows in other areas.

## Conclusions

Overexploitation of green turtle populations over the past several centuries has led to a shifting in the baseline of what is considered natural for seagrass ecosystems. Green turtle grazing in seagrass pastures is the natural condition. With successful conservation leading to increasing green turtle abundance, albeit still below historical numbers^16,17^, it is important to understand how seagrass ecosystem carbon dynamics will be affected. Seagrass meadows are important sites of blue carbon sequestration and storage^4,42^, and there is concern these functions may be affected as increased grazing pressure returns more seagrass meadows to their natural grazed state^22,43^. Here we show, through an *in situ* seagrass manipulation experiment and measurement of areas naturally grazed by green turtles, that rates of metabolic carbon uptake are lower in grazed areas than ungrazed areas. These differences in NEP correspond to a reduction in the potential of the meadow to sequester blue carbon in the future. However, grazing did not affect the P:R ratio of the meadow on short- or long-term time scales, suggesting that even sustained green turtle grazing is not likely to lead to a loss of sediment carbon through remineralization. These findings indicate that as more tropical seagrass habitats are returned to a natural grazed state, rates of carbon uptake and contribution to the metabolic carbon sink will be lower, but there will not be a large metabolic release of current blue carbon stocks.

## Methods

### Site Description

This experiment was conducted in seagrass meadows within Grape Tree Bay on the north side of Little Cayman, Cayman Islands (19°41′48.0″ N, 80°03′33.5″ W) at the Central Caribbean Marine Institute during May through August, 2016. Turtle grass (*Thalassia testudinum*) was the dominant seagrass comprising the meadows with interspersed manatee grass (*Syringodium filiforme*) and small amounts of shoal grass (*Halodule wrightii*) in some areas. The benthic habitat was comprised of carbonate sediments. The meadow was located roughly 40 m from shore in shallow water with a mean depth of 1.0 m and small tidal variation (±0.2 m). Mean height of the seagrass canopy was 15.8 cm with a mean *T. testudinum* density of 840 shoots m^−2^. Areas that were naturally grazed by green turtles for at least a year were present nearby in Grape Tree Bay. Naturally grazed areas had a mean *T. testudinum* blade length (canopy height) of 1.9 cm and a mean density of 776 shoots m^−2^.

### Experimental Design

We conducted an *in situ* clipping experiment to simulate grazing by green turtles. Ten 2 × 2 m plots were set up in an ungrazed area of the seagrass meadow. Five plots were experimentally clipped to simulate green turtle grazing, and five remained unclipped to serve as reference plots. All variables were measured in both clipped and reference plots prior to the onset of clipping to ensure that any changes measured were due to simulated grazing, and not previous differences. Prior to any measurements, the seagrass rhizomes were severed around the edges of all plots using a flat-bladed shovel to prevent the translocation of nutrients into the experimental plots from the surrounding unclipped meadow^10^. This was done to simulate a grazed area larger than 2 × 2 m, which is at the smaller end of the size range of natural grazing plots^44–47^. Reference plots were also severed to ensure that any effects seen in the clipped plots were the result of clipping, and not from severing rhizomes. All measurements were made and samples collected from the central 1.5 × 1.5 m of each plot so as to leave a 25 cm buffer zone around plot edges to avoid edge effects.

Clipping was initiated in May 2016 and maintained for twelve weeks. Clipped plots were initially established by clipping all blades within a plot just above the blade/sheath junction using scissors and collecting all clipped portions of the blades. Blades were re-clipped when blade length in the plot was ~5 cm above the blade/sheath junction to mimic natural turtle grazing^10,47^. This method resulted in clipping every ~14 days (range 12–15).

### Seagrass Measurements

Seagrass species composition and shoot density were measured bi-weekly in all plots. Data were collected from three randomly placed 25 × 25 cm (0.0625 m^2^) quadrats within each plot. Aboveground biomass samples were collected bi-weekly in clipped plots and monthly in reference plots from three 10 × 10 cm (0.01 m^2^) quadrats in each plot by clipping all shoots within the quadrat at the sediment surface. In the lab, blades were measured for length and width, gently scraped with a razor blade to remove any epiphytes, and rinsed in seawater. Samples were then dried at 60 °C for at least 24 hours before weighing for dry mass.

Belowground biomass samples were collected at the beginning and end of the experiment. Samples at the beginning were collected adjacent to the ten experimental plots to avoid destructive sampling within plots, and samples at the end were collected from the middle of each plot. Samples were collected using cylindrical PVC sediment cores (7.7 cm diameter) sharpened at one end and hammered into the sediment. All cores were deep enough to completely penetrate through the root/rhizome mat, resulting in a complete belowground biomass sample. Samples were refrigerated following collection and then processed within 48 hours. All aboveground biomass was removed from samples. Samples were cleaned of sediments using running seawater and then dried at 60 °C for at least 48 hours or until completely dry. Any remaining sediments were then removed, and samples were re-dried (60 °C) before weighing for dry mass.

These same methods were used for measuring aboveground biomass, belowground biomass, and seagrass parameters in the naturally grazed and ungrazed areas. Rhizomes were not severed in the naturally grazed or ungrazed areas as had been done around the edges of our experimental clipped and reference plots.

### Ecosystem Metabolism Measurements

Seagrass NEP and R_E_ measurements were made weekly using benthic incubation chambers. Sampling was prevented during weeks two and ten due to hazardous weather from tropical storms Colin and Earl. Chambers were comprised of a gas-tight, polyethylene bag with a sampling port attached to a rigid PVC cylinder^37,48,49^. PVC cylinders (16 cm inner diameter, encompassing 0.02 m^2^ of bottom area) were sharpened at one end and inserted roughly 7.5 cm into the sediment (not severing rhizomes)^37,48^. These were inserted in the sediment one day prior to the incubations to allow effects of the disturbance to dissipate prior to the incubation^50^. The polyethylene bags were then attached to the PVC cylinders with elastic bands and hose clamps. The use of a flexible bag allowed the propagation of turbulence to the interior of the chamber allowing internal mixing to more accurately mimic natural conditions^51^. Chamber volume was measured in the lab to be 5.5–6.0 L.

NEP and R_E_ were calculated from the change in dissolved oxygen (DO) concentration following an incubation period using light and dark (opaque) incubation chambers, respectively. Three water samples were collected in 60 ml plastic syringes (light or dark depending on the chamber) at the beginning and end of the incubation period from each chamber. Syringe samples were capped and returned to the surface immediately following collection, and DO measurements were taken directly in the syringe using a handheld optical DO meter (YSI ProODO), which was calibrated in water-saturated air on the morning of each sampling day^52^. We tested this method in the laboratory under various conditions prior to the field study to test and confirm the reliability of the method and the precision of YSI ProODO meters for this application. Mean incubation length in the field was 2.3 hours (range 1.5–3.3 hrs), as longer incubation periods may underestimate metabolic rates^41^. Incubations were always started by 1130 hours in order to encompass solar maximum. There were two instances in which a dark chamber gained oxygen during the incubation, suggesting an error. The gain in O_2_ during both of these instances was small, and within the margin of error of the DO probe, so these rates of respiration were assumed to be zero.

Water column metabolism was measured in a similar manner using clear and dark 300 ml BOD bottles. Three clear and three dark bottles were filled underwater at seagrass canopy height, capped, and attached to a PVC rod to incubate under *in situ* conditions. Three water column samples were collected in 60 ml plastic syringes at canopy height at the same time to measure initial water column DO concentration for the bottle incubations. One 60 ml plastic syringe was then collected from each BOD bottle following the incubation to measure final DO concentration. During instances when a dark bottle gained oxygen during the incubation (suggesting sampling error) DO changes were assumed to be zero. BOD bottle metabolic rates were subtracted from chamber rates to correct for water column metabolism and ensure that only metabolic rates of the benthic community were measured^53^. Water column metabolism played a minor role in this seagrass system. On average it contributed <5% of total reference plot metabolism within benthic chambers; however, it played a larger role on the two overcast days.

In addition to the experimentally clipped and reference plots, NEP and R_E_ were also measured using these same methods in nearby areas that were naturally grazed by green turtles (>1 year) and adjacent ungrazed areas of meadow. Three light and three dark incubation chambers were used on three occasions to measure NEP and R_E,_ respectively, in both naturally grazed and ungrazed areas.

### Data and Statistical Analyses

Hourly rates of NEP and R_E_ were calculated from changes in DO concentration measured in light and dark incubation chambers, respectively. Hourly GPP was calculated as the sum of NEP and the absolute value of R_E_. Daily rates were calculated by multiplying GPP by the photoperiod (10 hours) and R_E_ by 24 hours, and daily NEP was calculated as the difference between daily GPP and R_E_. Since all incubations were conducted during the middle of the day, we corrected length of daylight (13 hours) for dawn and dusk hours by assuming minimal production during the 1.5 hours on either end of the daylight period. We based estimates of GPP on the central 10 hours of daylight when solar irradiance values (HOBO Pendant data loggers) were comparable to those measured during our incubations. We assumed daytime and nighttime R_E_ to be equal for our calculations, and a deviation from this could result in a slight change in our calculated daily metabolic rates. Oxygen units were converted to molar units, and then converted to carbon units using photosynthetic and respiratory quotients of one^51^. While we measured ecosystem metabolism during the summer season, previous studies have shown that seagrass ecosystems are typically net autotrophic (NEP > 0) across the annual cycle, possibly becoming heterotrophic for only one to a few months per year (e.g. see references)^53-55^. Additionally, our study was conducted in a shallow, tropical location where water temperatures and incident sunlight do not vary greatly across seasons. We therefore do not feel that the conclusions of this study would be qualitatively different had we conducted the experiment for a full annual cycle.

The amount of aboveground seagrass biomass contained within incubation chambers was calculated by interpolating between clipping events. Assuming aboveground biomass to be zero immediately following clipping, we calculated daily biomass production rates using the aboveground biomass measured at time-of-clipping and the number of days since the previous clipping event. Using this calculated daily rate of biomass production, we estimated what mean areal aboveground biomass was for each plot during incubations based on how many days had elapsed between the incubation and the previous clipping event.

All calculations and statistical analyses were performed in R version 3.3.2^56^, and using the ‘reshape2’^57^ and ‘nlme’^58^ packages. The effect of simulated grazing on seagrass meadow GPP, R_E_, and NEP over time was evaluated using a mixed effects model with treatment and time as fixed factors and individual plot as a random factor. Linear regression was used to evaluate the relationship between GPP and R_E_ (production to respiration ratio) as well as seagrass meadow metabolic rates (GPP, R_E_, and NEP) with aboveground seagrass biomass. Unpaired t-tests (two-tailed), were used to evaluate differences in seagrass characteristics and aboveground biomass between clipped plots and naturally grazed areas. A one-way ANOVA was used to evaluate differences in the production to respiration ratio among treatments. A two-way ANOVA was used to evaluate differences in belowground seagrass biomass among treatments, with treatment and time as factors.

### Literature Collection of Seagrass Metabolic Rates

We searched the scientific literature for available estimates of seagrass metabolic rates to compare to those from this study by searching ISI Web of Science in October 2016 using the search terms: “seagrass metabolism” OR “seagrass metabolic” OR “seagrass production” OR “seagrass productivity.” Search results were further supplemented with the authors’ personal literature collections and citations therein.

Only results from *in situ* measurement of whole-system seagrass metabolic rates were extracted. Mesocosm studies and metabolic measurements from individual seagrass shoots were excluded. Additionally, only values from unmanipulated seagrass were used—if a manipulation was conducted (e.g. experimental shading), then only results from the control/reference treatment were extracted. In cases when multiple measurements were made within the same site (spatially or temporally) in a study, the mean was calculated for these replicates to obtain a single set of metabolic rates (GPP, R_E_, NEP) for each site.

Duarte *et al*.^42^ compiled a database of global seagrass metabolic rates, and data from this database were also used to supplement the values we extracted from the literature. All values obtained from the Duarte *et al*.^42^ database were confirmed in their original publication. In a few cases we were unable to obtain the original publication, and since we were unable to confirm these values or their method of collection they were excluded. Unpublished data, and data from studies that did not meet our requirements above were excluded.

This resulted in 58 unique estimates of seagrass metabolic rates—54 from the literature, and four new values from our study. Thirty-eight of these estimates come from studies included in the Duarte *et al*.^42^ database, and we have compiled an additional 20 estimates of seagrass metabolism here. All data extracted from literature sources or the Duarte *et al*.^42^ database were recorded in their reported units, and then converted to units of mmol C m^−2^ d^−1^ if needed. Values reported in oxygen units were converted to carbon units using photosynthetic and respiratory quotients of one.

Not all studies from which values were obtained used the same methods for measuring metabolic rates. While the vast majority of studies used *in situ* incubation chambers, bell jars, or the diel O_2_ curve method to measure seagrass metabolic rates, the length of time used for incubations or measurement varied among studies. It has been demonstrated that measured metabolic rates can be strongly affected by incubation length, and that longer incubations (e.g., 12 or 24-hour) tend to underestimate rates due to oxygen saturation or depletion within the chamber^41^. It is therefore possible that some seagrass metabolic rates reported in the literature are low due to methodological reasons, and the true rates in these systems may be higher than those reported.

### Data Availability

The compiled database of seagrass metabolic values collected from the literature (Supplementary Data 1) as well as the seagrass ecosystem metabolic data and seagrass biomass and morphometry data from this study (Supplementary Data 2) are available as supplementary information files.

## Electronic supplementary material

Supplementary Dataset 1

Supplementary Dataset 2
